# Prevalence of Oral Complications occurring in a Population of Pediatric Cancer Patients receiving Chemotherapy

**DOI:** 10.5005/iD-iournals-10005-1428

**Published:** 2017-06-01

**Authors:** Kapil Gandhi, Geetika Datta, Shilpa Ahuja, Tanvi Saxena, Ankush G Datta

**Affiliations:** 1 Professor, Department of Pedodontics and Preventive Dentistry Inderprastha Dental College & Hospital, Ghaziabad, Uttar Pradesh, India; 2 Reader, Department of Pedodontics and Preventive Dentistry Inderprastha Dental College & Hospital, Ghaziabad, Uttar Pradesh, India; 3 Senior Lecturer, Department of Pedodontics and Preventive Dentistry Inderprastha Dental College & Hospital, Ghaziabad, Uttar Pradesh, India; 4 Senior Lecturer, Department of Pedodontics and Preventive Dentistry Inderprastha Dental College & Hospital, Ghaziabad, Uttar Pradesh, India; 5 Ex-Senior Lecturer, Department of Orthodontics and Dentofacial Orthopedics Inderprastha Dental College & Hospital, Ghaziabad, Uttar Pradesh, India

**Keywords:** Chemotherapy, Malignancy, Oral complications, Pediatric.

## Abstract

Multiagent chemotherapy, radiotherapy, or a combination of both are the contemporary methods of cancer treatment. With medical advancements, though cure rates have increased considerably, focus is now shifted to the potential early and late complications of the same. The aim of this study was to assess the early oral complications in pediatric patients receiving chemotherapy. Sixty-two children with cancer undergoing chemotherapy with the mean age of 7.42 ± 3.6 years were included in the study. The various types of malignancies and oral problems during chemotherapy were recorded in the subjects. The most commonly encountered malignancy was acute lymphoblastic leukemia at 35.5%. Various oral and associated complications like mucosal inflammation with ulcerations, oral pain, xerostomia, and secondary infections were commonly seen, with mucositis being the most commonly observed complication in 58.1% of the subjects undergoing chemotherapy. Clinical importance of timely medical and dental interventions by a multidisciplinary team involving a pediatric dentist at different stages of anticancer treatment is also emphasized to minimize discomfort, increase treatment compliance, and improve the quality of life of pediatric patients.

**How to cite this article:**

Gandhi K, Datta G, Ahuja S, Saxena T, Datta AG. Prevalence of Oral Complications occurring in a Population of Pediatric Cancer Patients receiving Chemotherapy. Int Int J Clin Pediatr Dent 2017;10(2):166-171.

## INTRODUCTION

Thriving on more than 10 million patients worldwide, cancer has definitely surfaced as a major public health issue.^1^ In modern times, cancer has been widely diagnosed in children even though it is rarely considered as a childhood disease. Cancer is increasingly becoming a cause of death of children aged between 1 and 14 years; also the incidence of cancer is amounting to an average increase of 1% every year.^2^ The research shows that 1.6 to 4.8% of all cancers in India is diagnosed in children aged below 15 years; however, the overall incidence of 38 to 124 per million children per year is lower than that in the developed world.^3^ Despite the technological advancements in diagnosis and treatment of this disease, the plight of cancer does not seem to cease in the near future.

The developing countries are plagued by peculiar problems like population increase, lack of education, poverty, poor oral hygiene, and multiple health problem that contribute to higher occurrences of cancer. Eighty-five percent of pediatric cancer cases are concentrated in developing countries.^4^ Studies show that instances of cancer are more common in male child than in female child, with the male to female ratio being around 1.2:1 in most of the resource-rich countries.^5^ In India, the most common cancer affecting children is leukemia, with the relative proportion varying from 25 to 40%. Out of the reported leukemias, 60 to 85% are cases of acute lymphoblastic leukemia (ALL).^3^

With accomplished advances in the treatment of childhood cancer, there is an increase in young population who are successfully cured of their disease with the aid of various chemotherapeutic agents. With time, focus has shifted to mitigation of early and long-term side effects of several available treatment modalities.^6^

Ideally, a chemotherapeutic agent must completely erase all malignant cells. However, unavailability of anticancer drugs with such sparing effects has resulted in some damage to normal tissue. This can be particularly found in those tissues where rapid cell division normally occurs, such as oral and intestinal mucosa, bone marrow, hair follicle, liver, and testis.^7^

An array of significant therapy-related factors, viz., the type of chemotherapeutic agents used for therapy, the total dosage of the drug used, the frequency of drug administration, and other treatment modalities, such as radiotherapy used concomitantly with chemotherapy, affect the development of stomatotoxicity.^8,9^ Oral cavity is frequently affected with complications from cancer, or secondary cancer treatment. There has been substantial variety in the literature of reported incidences of such complications; however, it has been noted that children are most commonly affected by these complications.^10^ The factors affecting the severity of these complications include age of child, type of malignancy, condition of oral cavity before treatment, and the level of oral care during anticancer therapy.^11,12^

The common oral side effects include oral mucositis, intraoral infections, dry mouth, salivary gland inflammation, intraoral hemorrhage, and mucosal bleeding.^13^ Considering these factors and lack of existing information, a study was designed to investigate the prevalence and nature of orodental problems occurring in a population of pediatric cancer patients receiving chemotherapy in the city of Indore.

## MATERIALS AND METHODS

Sixty-two children between the age of 1 and 14 years who are receiving chemotherapy for malignancy and had complete treatment records in different hospitals treating pediatric cancer patients across Indore in Madhya Pradesh state were randomly selected and included in the study. Exclusion criteria included the children who were suffering from oral cancer and also children who were uncooperative. Data were collected on the basis of age, gender, malignancy type (solid tumor or hematologic malignancy), and chemotherapeutic drug administered (type, dose, and number of chemotherapeutic cycles) from hospital records.

Current blood data with particular attention to the white blood cell count, the platelet count (PC), and the hemoglobin level were recorded. Patient complaints, such as dry mouth and difficulty in swallowing, mastication, or in speech and pain were recorded.

Informed consent was secured from the parents and the treating oncologist prior to the examination of the patients. Patients were examined using sterile equipment and material, under day light or torch light as they are lying or sitting on the bed. The examination was conducted by a team of two examiners, which consisted of a trained doctor and an assistant. The examination was done using latex gloves, plain mouth mirror, probes, periodontal probes, tweezers, containers, cotton/gauze to remove any food debris and to dry the mucosa.

Scoring of chemotherapy-induced oral mucositis was done according to the World Health Organization (WHO) classification: Grade 0: No change; grade I: Soreness/ erythema; grade II: Erythema and ulcers, patient can eat solids; grade III: Ulcers, the patient requires liquid diet only; and grade IV: Food intake is not possible.^14^ Intraoral charting of teeth and their status was done. The decayed, missing, and filled teeth per subject (DMF) for primary and for permanent dentition were recorded as per the WHO criteria.^15^ Oral hygiene status was evaluated as per Simplified Oral Hygiene Index given by Greene and Vermillion.^16^

Both extraoral and intraoral clinical examinations were done. Any enlargement or tenderness in the sub-mental, submandibular, anterior and posterior cervical, and pre- and postauricular lymph nodes was recorded. The temporomandibular joints (TMJs) were palpated for any signs of pain or dysfunction. Intraorally, record of caries, gross tooth mobility, and tooth/jaw pain was made. Buccal and sulcular mucosa, the tongue, the floor of the mouth, the hard and soft palate, the fauces, and free and attached gingiva were examined systematically for evidence of any abnormality.

All the clinical data based on the examination of the patient were collected from the patients without distressing them and their attendants. Only the parents, attendants of the patients who wilfully provided consent for the examination and utilization of prevalent personal medical data from the hospital for the research work were included in the study and their written informed consent was recorded prior to the examination. No patient was subjected to any investigation for the study purposes.

## RESULTS

**Graph 1:**
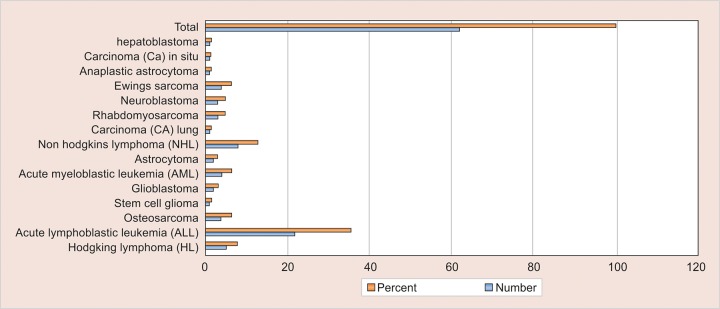
Distribution of malignancy in pediatric cancer patients

Thirty-four males and 28 females, between the age of 2 and 14 years, were included in the study group. Their mean age was 7.42 ± 3.619 years. The various types of malignancy and the number of subjects suffering from each type are shown in Table 1 and Graph 1.

**Table 1:** **Table 1:** Types of malignancy in the pediatric cancer patients

*Type of malignancy*		*Number*		*Percent*	
Hodgkin’s lymphoma (HL)		5		8.1	
Acute lymphoblastic leukemia		22		35.5	
Osteosarcoma		4		6.5	
Stem cell glioma		1		1.6	
Glioblastoma		2		3.2	
Acute myeloblastic leukemia		4		6.5	
Astrocytoma		2		3.2	
Non-Hodgkin’s lymphoma (NHL)		8		12.9	
Ca lung		1		1.6	
Rhabdomyosarcoma		3		4.8	
Neuroblastoma		3		4.8	
Ewing’s sarcoma		4		6.5	
Anaplastic astrocytoma		1		1.6	
Ca *in situ*		1		1.6	
Hepatoblastoma		1		1.6	
Total		62		100.0	

**Fig. 1:**
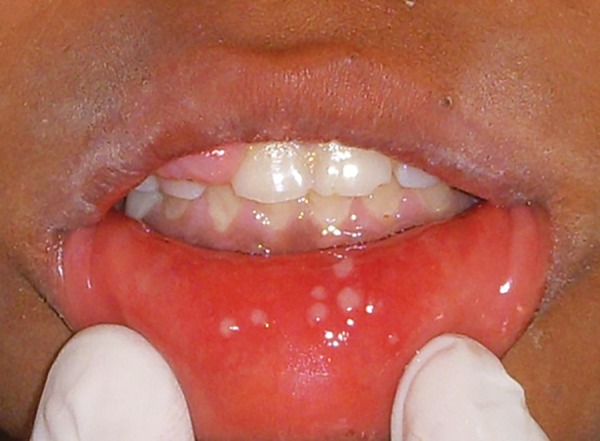
Mucositis of lips with ulcers

**Fig. 2:**
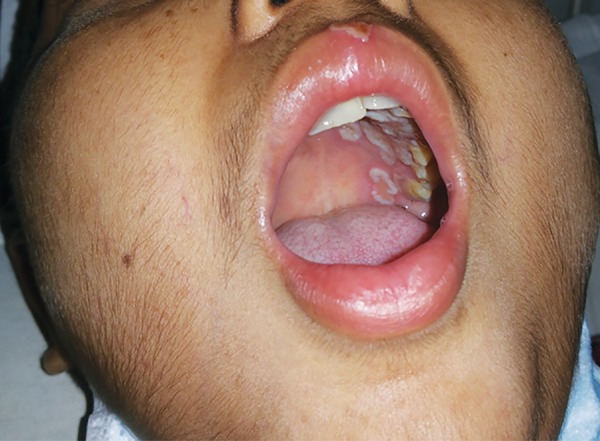
Ulcers on palate and lips

The most commonly encountered malignancy was ALL (35.5%), followed by non-Hodgkin’s lymphoma (12.9%), then Hodgkin’s lymphoma (8.1%), and Ewing’s sarcoma (6.5%). However, the other types like neuroblas-toma, hepatoblastoma, carcinoma lung were not frequent (Table 1). Various oral complications presented themselves coexisting with other complications in the subjects receiving chemotherapy (Table 2). On examination, oral mucosal ulcers and mucositis were the most frequently seen oral problems to occur during chemotherapy (Table 3). Out of 62 total subjects, 36 subjects suffered from varying degrees of oral mucositis. Clinically, symptoms observed varied from mild inflammation and redness of the mucosa to ulceration, with severe pain and bleeding (Fig. 1). Around 38.8% of patients had grade II mucositis while 8.3% of subjects had grade IV mucositis, which can lead to difficulty in oral food intake, severe pain, and at times necessitates the use of supplemental parenteral nutrition (Table 3 and Fig. 2). Ulcers were observed in 31 children and tended to appear 5 to 10 days after the start of chemotherapy. Secondary infection by *Candida* was seen in about five cases of mucosal ulceration in our study.

**Table 2:** **Table 2:** Prevalence of oral and associated complications

*Problem*		*No. of patients*		*Percent*	
Head and neck lymphadenopathy		*7*		11.2	
Lip cracking		8		12.9	
Oral mucosal ulcers		31		50	
Herpes simplex infection		6		9.7	
Temporomandibular joint pain		4		6.5	
Oral mucosal petechiae		14		22.6	
Ecchymosis		3		4.8	
Mucositis		36		58.1	
Oral pain		27		43.5	
Gingivitis		24		38.7	
Candidiasis		10		16.1	
Xerostomia		22		35.5	

**Table 3:** **Table 3:** Scoring of oral mucositis

*Oral mucositis*					
*Subcategories*		*n*		*%*	
Grade I		11		30.5	
Grade II		14		38.8	
Grade III		8		22.2	
Grade IV		3		8.3	

A significant proportion of subjects suffered complications related to bacterial and viral infections. Acute pseu-domembranous candidiasis occurred in 10 patients, while 6 patients developed herpes simplex virus (HSV) infections. Four cases of HSV infection were seen in patients with ALL. One patient of Hodgkin’s lymphoma and one of osteosarcoma suffered from HSV infection. Four subjects had localized suppurative dental infection suggestive of bacterial cause. Thrombocytopenia presented as oral petechiae, especially on soft palate and buccal mucosa. Ecchymosis was observed in about three patients where PC was below 20,000 mm^3^.

## DISCUSSION

Antineoplastic therapy in the form of radiotherapy and/ or chemotherapy can lead to an array of oral side effects which appear more frequently in the younger popula-tion.^17,18^ Systemic spread of pathogenic microorganisms can occur from the oral cavity through breach in the oral mucosal integrity induced by cytotoxic treatment that targets rapidly dividing cells.^19^ Any odontogenic infection can act as foci of sepsis which can cause serious life-threatening condition during periods of immune suppression.^20^ Marques and Walker^21^ have reported a case of severe facial cellulitis and secondary airway complication in the immunocompromised child due to a mobile exfoliating deciduous teeth.

Mucositis, aphthous ulcerations, secondary fungal or candida infections, xerostomia, oral pain, poor oral hygiene, gingival bleeding, and sialadenitis are some of the chemotherapy-induced oral complications manifesting in varying forms and degrees of severity. Such changes in the oral cavity can be painful, cause discomfort due to difficulty in eating and drinking, hence, interfering with and reducing treatment compliance. Necessary oral care at various stage of cancer treatment can prevent or reduce the incidence and severity of these oral compli-cations.^22^ Late complications of the oncology treatment may include altered dental development and craniofacial changes that can cause severe cosmetic and functional sequelae requiring surgical and orthodontic interventions in the future.^23^

Most commonly seen nonhematologic complication of cytotoxic chemotherapy is oral mucositis.^22^ In the present study, 58% of the total subjects suffered from varying degrees of oral mucositis. Guggenheimer et al^24^ and Jankovic et al^25^ reported similar results in their studies, with 52 and 55% respectively, of the cancer patients suffering from oral mucositis. An increased prevalence (69%) in pediatric cancer patients was observed in study by Wahlin and Matsson.^26^ This inflammatory condition generally begins 3 to 10 days after chemotherapy and can persist for 3 weeks. It has been shown to peak at around 7 to 14 days, at which time it slowly resolves unless complicated by infection.^27,28^ Mucositis can be all encompassing clinically presenting as mild inflammation to severe ulcerations, pain, bleeding, dysphagia, inability to eat/chew, drink, and talk, malnutrition, fatigue, and risk of both local opportunistic and systemic infection.^29^ Compromised oral intake and nutritional status accompanied with ulcerative pain can result in weight loss as well.^29-31^ In the present study, oral mucositis was clinically classified using WHO criteria (Table 3).^14^ Out of 26 subjects who were diagnosed with hematological malignancies, 16 had mucositis (61%) as compared with 57% in subjects who had solid tumors, which is consistent with the findings demonstrated by Sonis et al^31^ that children with hematologic malignancies experience mucositis more frequently than those with solid tumors. Also certain chemotherapeutic drugs, such as doxorubicin, bleomycin, fluorouracil, and methotrexate and poor oral hygiene are associated with increased incidence of oral mucositis.^30,32^ The Multinational Association of Supportive Care in Cancer/International Society of Oral Oncology has published guidelines for the treatment of mucositis. The primary treatment of mucositis is palliative therapy, which includes home oral hygiene, pain control through use of analgesics or topical anesthesia, nonmedicated oral rinses (e.g., 0.9% saline or sodium bicarbonate mouth rinses 4-6 times/day), and symptomatic treatment of dry mouth.^33^ Recently, Palifermin, a keratinocyte growth factor-1 in patients undergoing high-dose chemotherapy, is being considered.^34^

Oral pain was the next frequent oral manifestation in cancer patients undergoing antineoplastic therapy; 43.5% of total subjects had pain in the oral cavity accompanied with mucosal ulcerations and mucosal inflammation in most of the cases. Twenty-two subjects out of 27 who complained of oral pain in our study were receiving vincristine as part of their treatment. McCarthy and Skill-ings^35^ reported similar findings in their study and stated that oral pain in the absence of a dental/periodontal infection can also be a neurotoxic side effect of vincristine and vinblastine. They observed that a detailed clinical and radiographic study is further required to distinguish such pain from pulpal pain.^17^ In a study done by Miser et al^36^ in 1987 on 139 children and young adults, the evaluation of treatment-related pain revealed the most common cause to be mucositis, followed by postoperative pain and neuropathic pain due to vincristine.^17^

Immune prematurity, weakened host defense mechanisms can increase the susceptibility of an already immunocompromised patient to infections commonly bacterial and fungal in origin. Oral fungal infections particularly *Candida* are seen in patients with prolonged, severe neu-tropenic episodes leading to systemic complications, such as fungal esophagitis or even septicemia. Broad spectrum antibiotics, steroids, or any preexisting fungal infection further contribute to the risk.^37,38^ Herpes simplex virus is a common pathogen in cancer patients, which can aggravate oral mucositis.^39^ In most cases, reactivation of latent virus can lead to infections from HSV, varicella-zoster virus, and Epstein-Barr virus, while cytomegalovirus infection can result from the reactivation of a latent virus or from a recently acquired virus.^17^ Bacterial orodental infections usually caused by Gram-negative organisms are seen. Reduced inflammatory response in periods of myelosuppression can mask the symptoms, further causing difficulty in diagnosis. Compromised host defense, mucosal inflammation, or disruption can lead to serious infections by *Streptococcus viridans,* which is a part of normal oral microflora.^37^ Consequently, it is important that oral hygiene protocols should be planned and followed to reduce the level of microbial colonization of the dentition and periodontium particularly during the period of bone marrow suppression. In the present study, bacterial infections were seen among four subjects. Close monitoring of the oral cavity allows for timely diagnosis and treatment of fungal, viral, and bacterial infections. Microbial surveillance of all suspicious lesions should be performed and prophylactic medications should be initiated until more specific therapy can be (g chemo) prescribed.^17,33^

The American Academy of Pediatric Dentistry recognizes that the pediatric dental professional plays an important role in the diagnosis, prevention, stabilization, and treatment of oral and dental problems in pediatric oncology patients as a part of the multidisciplinary team approach.^33^ Emphasis is given to the role of the dental care team which should primarily focus on early diagnosis and preventive treatment so as to reduce the risk of infection and minimize the adverse effects of anticancer therapies. A detailed medical history of each cancer patient including information about the nature of disease, treatment planned, any episode of relapse, or complications should be taken in collaboration with the oncology team. The current hematological status, systemic review, or existing/potential source of any oral/systemic infection should also be considered. Pretreatment dental evaluation aims at recognizing and removal of any active and potential sources of infection in the oral cavity, possible sources of local irritation, such as a sharp tooth cusp or a rough area on a restoration, and oral cancer screening.^33,40^

## CONCLUSION

Antineoplastic therapy can have adverse effects on the oral health of a cancer patient, which in turn can profoundly affect the general well-being of the patient. Oral mucositis, xerostomia, and opportunistic infections are more common in pediatric patients. Early involvement of a pediatric dentist can ensure effective oral care parallel to the ongoing oncological treatment, thus making a recognizable difference in the treatment success and palliative care. Professionally planned oral hygiene protocols, regular follow-ups, oral microbial surveillance, and palliative care to reduce oral discomfort caused due to antineoplastic therapy can help patients and their parents in combating the distressing yet unavoidable side effects of antineoplastic therapy. Further studies need to be conducted to evaluate on larger population to establish a correlation between various chemotherapeutic agents and oral or perioral complications and pain.

## CLINICAL SIGNIFICANCE

Pediatric dental professionals are expected and play a key role in the multidisciplinary management of the pediatric cancer patients. The article aims to sensitize the pediatric dental professionals toward the management needs by highlighting the commonly encountered oral complications of such patients, which may vary according to the various chemotherapeutic agents used.
